# Upregulated 5-HT_1A_ receptor-mediated currents in the prefrontal cortex layer 5 neurons in the 15q11–13 duplication mouse model of autism

**DOI:** 10.1186/s13041-020-00655-9

**Published:** 2020-08-24

**Authors:** Fumihito Saitow, Toru Takumi, Hidenori Suzuki

**Affiliations:** 1 Department of Pharmacology, Nippon Medical School, 1-1-5 Sendagi, Bunkyo-ku, Tokyo, 113-8602 Japan; 2 RIKEN Brain Science Institute, Wako, Saitama, 351-0198 Japan; 3 Department of Physiology and Cell Biology, Kobe University School of Medicine, Chuo, Kobe, 650-0017 Japan

**Keywords:** 5-HT_1A_ receptor, Autism, Developmental disorder, Prefrontal cortex, Serotonin, Synapse

## Abstract

Serotonin (5-HT) is a well-known modulator of behavioral, physiological, and emotional functions of the forebrain region. We recently discovered alterations of serotonergic synaptic modulations in both, the prefrontal cortex (PFC) and the somatosensory cortex, in the *15q dup* mouse model of autism spectrum disorder (ASD). To further understand the roles of the 5-HT system implicated in developmental disorders such as ASD, comparison with model animals exhibiting different phenotypes may be useful. In this study, we investigated the relationship between sociability and the magnitude of 5-HT_1A_ receptor (5-HT_1A_R) activation-induced outward currents from layer 5 pyramidal neurons in the PFC, because a mouse model of Williams-Beuren syndrome (WBS; another developmental disorder exhibiting low innate anxiety and high sociability) reportedly showed larger 5-HT-induced currents. To investigate whether the 5-HT_1A_R activation-induced outward currents are involved in the endophenotype determination of social behavior, we examined *15q dup* mice with a phenotype opposite to WBS. We found 5-HT elicited significantly larger outward currents in *15q dup* mice than in WT controls, regardless of sociability. In contrast, baclofen-induced GABA_B_ receptor-mediated outward currents were not significantly different between genotypes, although GABA_B_ receptor was coupled to G_i/o_ as well as 5-HT_1A_. Further, we found the larger 5-HT_1A_R-mediated currents in *15q dup* mice did not affect the magnitude of inhibitory action of NMDA receptor functions. Taken together, our results provide a potential physiological hallmark for developmental disorders that may involve the imbalance of the neuronal circuity in the PFC.

## Introduction

Autism spectrum disorder (ASD) is a neurodevelopmental disorder characterized by three symptoms: deficits in social communication, delayed speech and impaired communication, and stereotyped and repetitive behaviors. The chromosome 15q11–13 duplication (*15q dup*) mouse, corresponding to cytogenetically frequent copy number variation in ASD, has core symptoms such as impaired social interaction and anxiety-like behaviors [1–4]. Therefore, *15q dup* mice retain part of the clinical and genetic features of ASD, and could be an appropriate model to investigate the pathophysiology of ASD. Since serotonin (5-HT) is a well-known modulator of behavioral, physiological, and emotional functions in the forebrain, our research has explored the role of serotonin in synaptic dysfunction in developmental disorders. Recently, we found that *15q dup* mice experienced a hyposerotonergic state in the brain, and a reduction in activity of the dorsal raphe nucleus (DRN) neurons. Further, we found that administration of the selective serotonin reuptake inhibitor (SSRI) fluoxetine during the early postnatal period normalized 5-HT levels in adulthood; in addition, impairments in electrophysiological properties of the DRN and social behavior were both ameliorated [5]. More recently, we found that impaired social behavior, one of the symptoms of ASD, has been linked to an altered balance between excitatory and inhibitory neurotransmission within the cortex, including the prefrontal cortex (PFC) [6, 7]. The observed results could be partially explained by differences in serotonergic modulation in the inhibitory interneurons present in both the sensory cortex and the PFC. Another developmental disorder, Williams syndrome (or Williams-Beuren syndrome; WBS), is associated with the opposite endophenotypes of social behavior to those seen in ASD. For example, one of the notable features of WBS is the high sociability and fearlessness with regards to other people [8, 9]. The homozygous deletion of general transcription factor *GTF2IRD1* (*Gtf2ird1*^*−/−*^) is used as an animal model of WBS [10]. According to a previous report by Proulx et al. [11], the magnitude of 5-HT_1A_ R activation-induced outward currents from the L5 pyramidal neurons in the PFC is enhanced in WBS model mice. Assuming that there was a correlation between the magnitude of the 5-HT_1A_R activation-induced outward currents and the expression of social behavior, we hypothesized that the current amplitude would be smaller in *15q dup* autism model mice.

Therefore, the first aim of this study was to determine whether the magnitude of the 5-HT_1A_R-mediated outward current could represent a criterion for the level of social behavioral abnormalities. However, contrary to our expectations, the amplitude of the 5-HT_1A_R-mediated outward currents in the *15q dup* model was larger than that seen in the wild type mice. Next, we examined whether the larger 5-HT_1A_R-mediated currents in the *15q dup* model affected inhibitory action on N-Methyl-D-aspartate receptor (NMDAR) function, since 5-HT_1A_R activation inhibits NMDAR-mediated currents in the PFC [12, 13]. However, between genotypes, no difference was found in the extent of the inhibition of NMDAR-mediated currents by 5-HT_1A_R activation. Given the 5-HT_1A_R-mediated inhibitory action of L5 pyramidal neurons, our results provide a potential physiological hallmark for developmental disorders that may involve an imbalance in the neuronal circuity of the PFC.

## Materials and methods

### Animals

All mice had a C57BL/6 J background and were kept under a constant temperature (25 ± 1 °C) and a regular light/dark cycle (lights on from 06:00 to 20:00), with free access to food and water. Paternal *15q dup* generation has been previously reported [1], and the line is maintained by mating male *15q dup* with WT female C57BL/6 J mice (Japan SLC Inc. Hamamatsu, Japan). All mice were weaned from 3 to 4 weeks post-birth and housed in same-sex groups (3–5 mice per cage). All experiments were conducted in accordance with the National Institute of Health Guide for the Care and Use of Laboratory Animals and were approved by the Institutional Animal Care and Use Committee of Nippon Medical School (approval number: 27–034).

### Electrophysiology

Brain slices for experiments were prepared from mice aged 9–12 weeks. The mice were deeply anesthetized with isoflurane inhalation (~ 4% in air, v/v) and then underwent trans-cardial perfusion with 10 mL of ice-cold carbogenated N-Methyl-D-glucamine (NMDG) cutting solution [14] that contained the following compounds: 110 mM NMDG, 30 mM NaHCO_3_, 2.5 mM KCl, 0.5 mM CaCl_2_, 10.0 mM MgCl_2_, 1.25 mM NaH_2_PO_4_, 12.5 mM glucose and 20 mM HEPES (adjusted pH of 7.4 with HCl). Following decapitation, the mice brains were rapidly removed and placed in NMDG cutting solution. The brain was blocked and three coronal slices (each 300 μm thick) were cut using a vibratome (VT1200s; Leica, Wetzlar, Germany) through the entire rostro-caudal extent of the PFC, between 2.34 and 1.54 mm from bregma, according to the atlas of the mouse brain [15], and placed in a submerged chamber for at least 1 h in artificial cerebrospinal fluid (ACSF) that contained 125.0 mM NaCl, 2.5 mM KCl, 2 mM CaCl_2_, 1.3 mM MgCl_2_, 26.0 mM NaHCO_3_, 1.25 mM NaH_2_PO_4_ 2H_2_O, and 11.0 mM glucose. ACSF was maintained at pH 7.4 by bubbling 95% O_2_–5% CO_2_ gas.

**Fig. 1:**
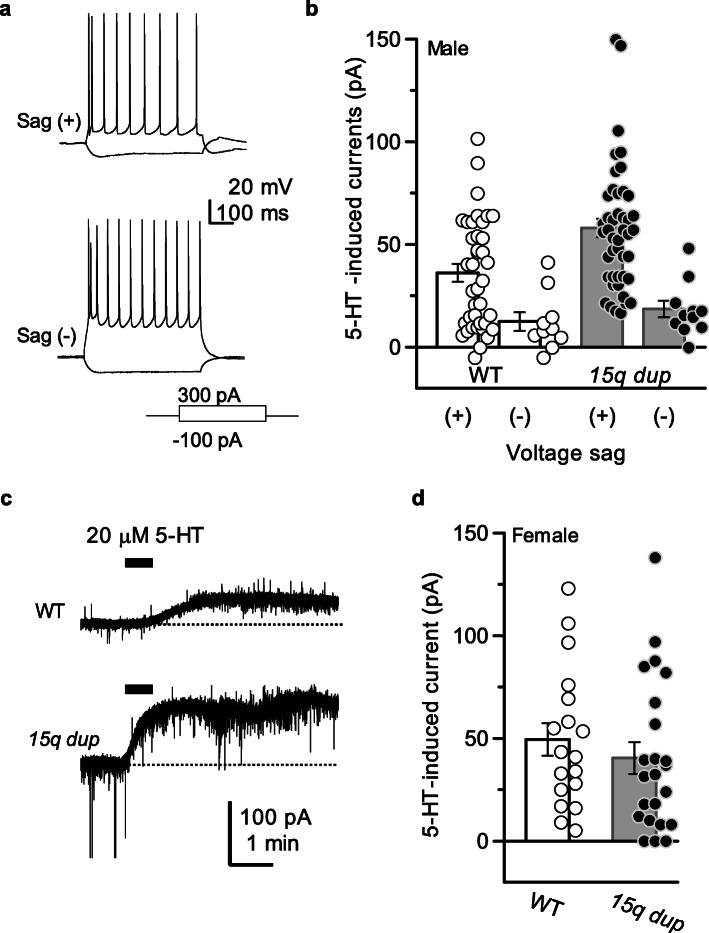
5-HT elicits a large outward current in the PFC L5 pyramidal neurons from 15q dup mice. **a** Electrophysiological differences in the membrane properties among L5 pyramidal neurons in the PFC. Upper traces (Sag(+)) were obtained from putative pyramidal tract neurons, while lower traces (Sag(−)) were obtained from putative intratelencephalic neurons in male mice. According to our criteria, more than 3 mV of the sum of the voltage sag and rebound depolarization with the current-injection of − 50 pA are defined as “Sag(+)” pyramidal neurons. **b** Representative traces of 5-HT-induced outward current obtained from male “Sag(+)” pyramidal neurons by bath-application of 5-HT (20 μM for 30 s). Bars indicate the timing of exogenous application of 5-HT. **c** The amplitudes of 5-HT-induced outward currents were neuronal type-dependent in male mice of both genotypes (WT: open circles; *15q dup*: filled circles). Sag(+) pyramidal neurons display significantly larger 5-HT-induced outward currents in the *15q dup* mice (WT: 36.18 ± 4.33 pA, *n* = 39; *15q dup*: 58.01 ± 4.53 pA, *n* = 45, t (101) = 4.31, *p* = 8.5 × 10^− 5^; Steel-Dwass multiple comparison test). On the other hand, Sag(−) pyramidal neurons between genotypes were not significantly different (WT: 12.44 ± 4.53 pA, *n* = 10; *15q dup*: Sag(−): 18.55 ± 4.02 pA, *n* = 11, *p* = 0.33; Steel-Dwass multiple comparison test). **d** Female mice Sag(+) pyramidal neurons displayed a similar extent of 5-HT_1A_ receptor-mediated currents (WT (open circles): 49.41 ± 7.98 pA, *n* = 22; *15q dup* (filled circles): 40.51 ± 7.68 pA, *n* = 17, t (39) = 0.79, *p* = 0.43). Bar graphs represent mean ± S.E.M.

Individual slices were transferred to a recording chamber attached to a microscope stage and continuously perfused with oxygenated ACSF at a flow rate of 1.4 ml/min, maintained at ~ 30 °C. Borosilicate glass-patch electrodes (World Precision Instruments, Sarasota, FL, USA) were used for whole-cell patch-clamp recordings from PFC L5 pyramidal neurons with a resistance of 2–3 MΩ when filled with an internal solution of 150.0 mM CH_3_KO_3_S, 5.0 mM KCl, 0.1 mM egtazic acid potassium salt [K-EGTA], 10.0 mM Na-HEPES, 3.0 mM Mg-adenosine triphosphate [ATP], and 0.4 mM Na-guanosine triphosphate [GTP] (pH 7.4). For electrical stimulation-evoked NMDAR-mediated excitatory postsynaptic current (NMDA-EPSC) recordings, we reduced the leak conductance of the membrane for voltage-clamping by using a cesium-based solution (150.0 mM Cs-methanesulfonate, 0.1 mM Cs-EGTA, 1.0 mM MgCl_2_, 20.0 mM Na-HEPES, 3.0 mM Mg-ATP, and 0.4 mM Na-GTP (pH 7.4)). Neurons in the PFC were visually identified under infrared differential interference contrast (IR-DIC) imaging by using a water-immersion objective (40X, NA = 0.80; Olympus, Tokyo, Japan). L5 pyramidal neurons in the PFC are divided into at least two subpopulations, which can be identified by their electrophysiological properties [16, 17]. After performing the whole-cell patch-clamp configuration, we identified the cell type by current injection under the current-clamp mode. Our neurons of interest in L5 are pyramidal tract neurons which elicit prominent voltage-sag and rebound afterdepolarization in response to hyperpolarizing current injections, as shown in Fig. 1a (upper traces, Sag(+)).The membrane properties of the recorded neurons could not be characterized using the Cs^+^-based internal solution, as shown in Fig. 3. However, the probability of encountering Sag (+)-type was relatively high (> 80%) [18]. Therefore, we considered Sag(+)-type neurons to be recorded in most cases (Fig. 3).

Whole-cell patch-clamp recordings were acquired and controlled using the Axon 700B Multiclamp amplifier (Molecular Devices, San Jose, CA, USA) and pClamp10 acquisition software (Molecular Devices). The cell characteristics of the membrane properties, including resting membrane potential and action potential properties, were measured in the current-clamp mode. For recordings of 5-HT-induced currents, the cells were voltage clamped at − 60 mV. Except for the experiments shown in Fig. 1d, data were obtained from Sag (+) type neurons of male mice. Obtained data were analyzed using Clampfit (Molecular Devices) and Kyplot 6 (Kyenslab, Tokyo, Japan) software.

### Compounds

We obtained 5-HT, gabazine, 6-cyano-7-nitroquinoxaline-2,3-dione (CNQX), D,L-2-amino-5-phosphonopentanoic acid (D,L-APV) and (±)-8-Hydroxy-2-(dipropylamino) tetralin hydrobromide (8-OH-DPAT) from Sigma-Aldrich (St. Louis, MI, USA); WAY100635 and baclofen from Tocris Bioscience (Bristol, UK); and tetrodotoxin from FUJIFILM Wako Pure Chemical (Tokyo, Japan).

### Data analysis

All data are expressed as means ± the standard error of the mean (SEM), with *n* representing the number of independent experiments. We evaluated statistical differences with parametric Student’s *t*-tests. For multiple comparisons between experimental groups, we used Steel’s nonparametric multiple comparisons test. A *p*-value of less than 0.05 was considered to indicate statistical significance. Statistical analysis was performed with Kyplot 6 software.

## Results

### 5-HT-induced outward currents in L5 pyramidal neurons is larger in *15q dup*

Whole-cell recordings were performed from L5 pyramidal neurons of the prelimbic region of the PFC. According to a study by Gee et al. [16], the level of hyperpolarization-activated cyclic nucleotide-gated cation (HCN) channels is useful to reliably separate the pyramidal tract and intratelencephalic neurons. During the course of the experiments, we rarely encountered recording of neurons with lower activity of HCN channels (intratelencephalic neurons), as shown in Fig. 1a (below traces, Sag(−)). On the other hand, pyramidal neurons displaying prominent voltage-sag and rebound depolarization with a hyperpolarization current (− 100 pA) could be frequently recorded (encounter rate: ~ 80%), as shown in Fig. 1a (upper traces, Sag(+)). In our experiments, more than 3 mV of the sum of the voltage sag and rebound depolarization with a − 50 pA current-injection are defined as “Sag(+)” pyramidal neurons. In both neuron types, bath application of 5-HT (20 μM, 30 s) elicited outward currents under the voltage-clamp condition. In both genotypes of male mice, 5-HT-induced outward currents were significantly larger in Sag(+) neurons (Fig. 1b, WT: Sag(+): 36.18 ± 4.33 pA, *n* = 39, Sag(−): 12.44 ± 4.53 pA, *n* = 10, t (101) = 2.77, *p* = 0.025; *15q dup*: Sag(+): 58.01 ± 4.53 pA, *n* = 45, Sag(−): 18.55 ± 4.02 pA, *n* = 11, t (101) = 4.31, *p* = 8.5 × 10^− 5^; Steel-Dwass multiple comparison test). These results are consistent with previous studies [19]. The mean 5-HT-induced outward current in the Sag(+) type was significantly larger in the *15q dup* mice (Fig. 1b and c, t(101) = − 3.35, *p* = 0.004; Steel-Dwass multiple comparison test) in the absence of differences in the basic membrane properties of the neurons, including the resting membrane potential (WT -68.82 ± 0.60 mV; *n* = 38, *15q dup* − 69.6 ± 0.57 mV; *n* = 43, t (79) = 0.94, *p* = 0.35; unpaired t-test), and the input resistance (WT 92.2 ± 4.79 MΩ; n = 38, *15q dup* 95.07 ± 3.89 MΩ; n = 43, t (79) = − 0.47, *p* = 0.64; unpaired t-test) in the PFC L5 pyramidal neurons. Moreover, it has been reported that 5-HT-induced outward current at the L2/3 pyramidal neurons of the PFC had sex-dependent difference in adult rats [20]. Therefore, we examined whether adult mice also exhibited a sex difference in the 5-HT-induced outward current in the Sag(+) type neurons at L5 of the PFC. As shown in Fig. 1d, there was no significant difference in the 5-HT-induced outward current amplitude in female mice (WT: 49.41 ± 7.98 pA, *n* = 22; *15q dup*: 40.51 ± 7.68 pA; *n* = 17, t (39) = 0.79, *p* = 0.43; unpaired t-test).

### 5-HT-induced outward currents in L5 pyramidal neurons are predominantly mediated by 5-HT_1A_ receptor activation

**Fig. 2:**
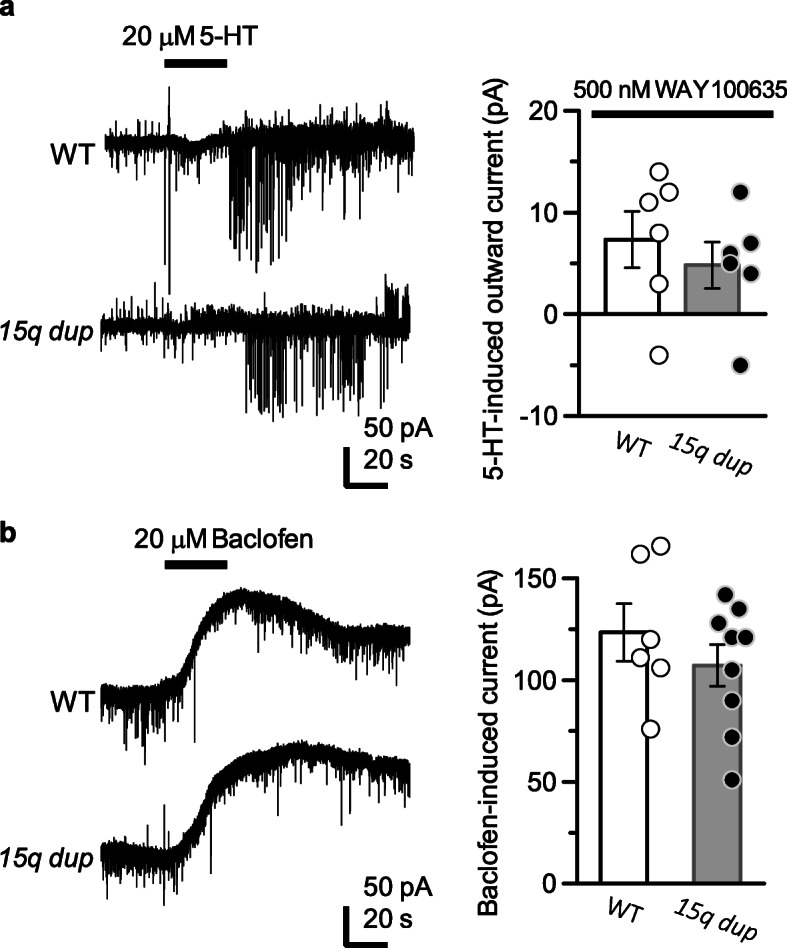
5-HT-induced outward currents are predominantly mediated by 5-HT_1A_ receptor activation, and other G_i/o_-linked GPCR activation-mediated outward currents were not changed in genotype. **a** In the presence of 5-HT_1A_ receptor antagonist WAY100635 (500 nM), 5-HT-induced outward current was largely suppressed (*Left trace*). There was no difference in the amplitude of 5-HT-induced outward current (WT: 7.33 ± 2.75 pA, *n* = 6; *15q dup*: 4.88 ± 2.27 pA, *n* = 6, t (10) = 0.70, *p* = 0.50; unpaired t-test). Bar graphs represent mean ± S.E.M. **b** Tests for other Gi/o-coupled receptor activation in both genotypes. GABA_B_ receptor agonist baclofen (20 μM for 30 s) elicited large outward currents (Left traces) and exhibited statistically similar responses in both genotypes (WT: 123.50 ± 14.17 pA, *n* = 6; *15q dup*: 107.22 ± 10.20 pA, *n* = 9, t (13) = 0.96, *p* = 0.36; unpaired t-test). Bar graphs represent mean ± S.E.M.

It is well-known that the main inhibitory action of 5-HT on the membrane of L5 pyramidal neurons mediates 5-HT_1A_R activation [21–23]. We therefore explored the development of a pharmacological test for 5-HT-induced outward current using a 5-HT_1A_ receptor antagonist, WAY100635 (WAY, 500 nM). In the presence of WAY, the 5-HT-induced outward current was largely suppressed, as shown in Fig. 2a. Between genotypes, there was no difference in the amplitude of 5-HT-induced outward current (WT: 7.33 ± 2.75 pA, *n* = 6; *15q dup*: 4.83 ± 2.27 pA, n = 6, t (10) = 0.70, *p* = 0.50; unpaired t-test). These results suggest that the 5-HT-induced outward currents are mainly mediated by 5-HT_1A_R activation. Further, ongoing responses under 5-HT_1A_R blocking might be due to the activation of 5-HT_5A_ receptors, based on the results of a previous study [24]. Therefore, it can be concluded that the contribution of 5-HT_5A_ receptors did not change between genotypes.

With regard to enhancing the downstream effectors of the 5-HT-induced outward current in *15q dup*, the currents elicited by other G_i/o_-coupled receptors may also be enhanced by the amplitude of the outward currents mediated by G-protein coupled inward rectifying potassium (GIRK) channels. In order to confirm this hypothesis, baclofen was used as an agonist for G_i/o_-coupled GABA_B_ receptors in accordance with a previous study [11] and compared the outward current elicited by baclofen (Fig. 2b). Both 5-HT- and baclofen-induced currents showed inward rectification, and these responses were largely inhibited by the non-specific GIRK channel blocker, barium (Ba^2+^: 200 μM; Supplementary Figure 1). Bath application of baclofen (20 μM for 30 s) induced large outward currents similar to those elicited by 5-HT in L5 pyramidal neurons. However, the amplitude of the outward currents were not significantly different between genotypes (WT: 123.50 ± 14.17 pA, *n* = 6; *15q dup*: 107.22 ± 10.20 pA, *n* = 9, t (13) = 0.96, *p* = 0.36; unpaired t-test). Together, these results suggest that the receptor function of 5-HT_1A_Rs is specifically enhanced in L5 pyramidal neurons of *15q dup* mice.

### Modulation of 5-HT_1A_R activation on NMDA receptor-mediated synaptic currents is not altered

**Fig. 3:**
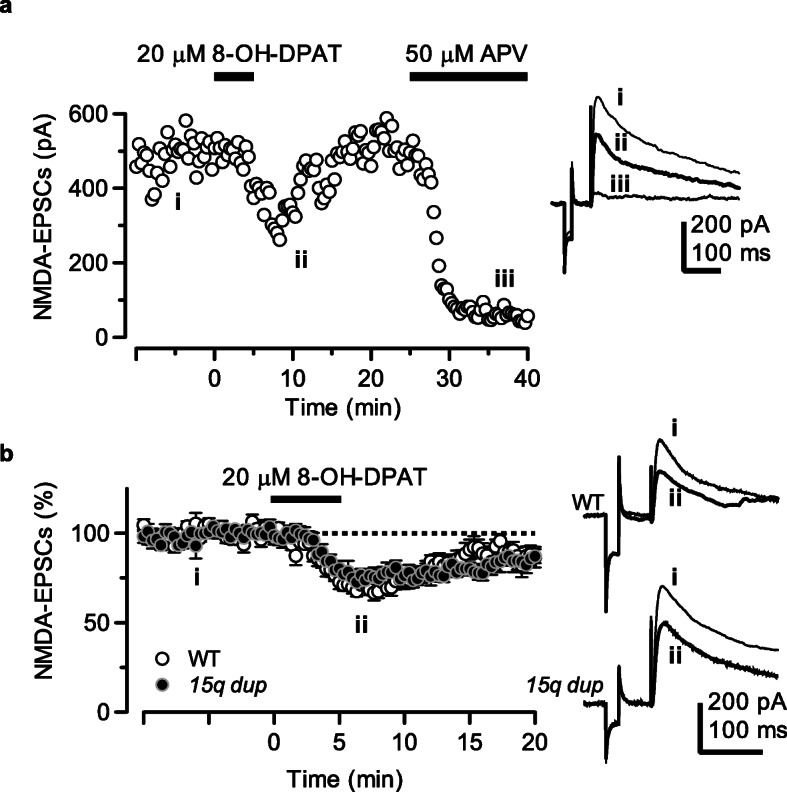
Extent of inhibitory action of NMDA receptor-mediated currents by activation of 5-HT_1A_ receptors did not affect L5 pyramidal neurons in the *15q dup* mouse model. **a** Representative time course of reversible reduction of NMDA-EPSCs by bath-application of the 5-HT_1A_ receptor agonist 8-OH-DPAT (20 μM for 5 min, trace ***ii***). At the end of the recording, EPSC amplitude was completely suppressed by the NMDAR antagonist APV (50 μM, trace ***iii***), indicating that the recorded synaptic current was mediated by the NMDARs. Right traces were obtained at the time points indicated in the graph of the left. Trace ***i*** indicates the baseline level of NMDA-EPSC. These recordings were conducted in the presence of gabazine (500 nM) and CNQX (10 μM) in order to block both GABA_A_- and AMPA/kainate-receptor mediated synaptic transmissions. **b** Effects of bath-application of 8-OH-DPAT on NMDA-EPSC amplitude at a holding potential of + 50 mV. The amplitude of NMDA-EPSCs was expressed as a percentage of the baseline (determined for 10 min before application of 8-OH-DPAT). The inhibitory action on NMDA-EPSCs by 8-OH-DPAT was similar in both genotypes at 5–7 min (WT (open circles): 71.36 ± 2.96%, *n* = 12; *15q dup* (filled circles): 75.75 ± 3.90% *n* = 14, t (24) = − 0.87, *p* = 0.39; unpaired t-test). Each symbol represents mean ± S.E.M. Right traces were shown as representative recordings. The traces shown are the average of eight consecutive sweeps recorded at the time points indicated in the time course of the left graph

Previous studies by Yuen et al. revealed that the activation of 5-HT_1A_Rs exhibited an inhibitory action on the NMDAR-mediated current amplitude in PFC pyramidal neurons [12, 13]. NMDARs are involved in various neural functions such as synaptic plasticity [25], and their dysfunctional state is associated strongly with the pathophysiology of mental disorders, including schizophrenia and major depressive disorder [26–28]. Therefore, we tested whether 5-HT_1A_R activation elicited a more inhibitory effect on NMDAR-mediated synaptic responses in *15q dup* mice, since the 5-HT_1A_R-mediated current responses were significantly larger in *15q dup* mice. To examine the modulatory action of NMDARs, we used a cesium-based internal solution for improving a space clamp with holding potential at + 50 mV. In the presence of both the GABA receptor antagonist gabazine (500 nM) and the AMPA / kainate receptor antagonist CNQX (10 μM), electrical stimulation-evoked NMDAR-mediated synaptic currents (NMDA-EPSCs) onto pyramidal neurons could be recorded as outward currents, as shown in Fig. 3a (trace **i**). Bath-application of 5-HT_1A_R agonist 8-OH-DPAT (20 μM, 5 min) reversibly decreased the amplitude of NMDA-EPSCs (Fig. 3a (trace **ii**)), and these synaptic currents were completely suppressed by the NMDAR antagonist APV (50 μM; Fig. 3a (trace **iii**)). These effects of 8-OH-DPAT on both the extent and the time course of NMDA-EPSC suppression were consistent with previous observations [12, 13]. When comparing between genotypes, contrary to our expectations, no difference was found in the extent of the inhibitory effect on NMDA-EPSCs at 5–7 min after application of 8-OH-DPAT (Fig. 3b; WT: 71.36 ± 2.96%, *n* = 12; *15q dup*: 75.75 ± 3.90% *n* = 14, t (24) = − 0.87, *p* = 0.39; unpaired t-test). 5-HT only influenced the generation of 5-HT_1A_R-mediated larger outward currents, but did not result in significant inhibition of the function of NMDARs through the activation of 5-HT_1A_Rs in *15q dup* mice. Together, these results suggest that the downstream signals of 5-HT_1A_R activation have distinct pathways to generate the outward currents and the inhibition of NMDARs in L5 pyramidal neurons of the PFC.

## Discussion

In this study, we found that L5 pyramidal neurons in the PFC elicited significantly larger outward currents in the *15q dup* mouse model of autism, compared to WT mice. As per our original hypothesis, we expected a correlation between innate sociability levels and the extent of 5-HT_1A_-mediated outward currents. However, our results were surprisingly similar to the results from a previous report using a mouse model of WBS, which exhibited a low anxiety, low aggression and high sociability phenotype [11]. Given that anxiety-like behaviors [1, 2, 29] and impairment of social behaviors [1, 3, 5] were observed in *15q dup* mice, the phenotype of *15q dup* mice is opposite to that of the WBS model mice. Therefore, the enhancement of 5-HT_1A_R-mediated outward currents is considered to be a pathophysiological feature commonly found in developmental disorders with independent phenotypes such as anxiety and sociability levels. However, 5-HT_1A_R-mediated outward currents in female mice did not differ between genotypes. A previous study [20] has shown that the amplitude of 5-HT_1A_R-induced currents in young rats of both sexes exhibited relatively large responses. Although the amplitude of 5-HT_1A_R-mediated currents in females showed only a small reduction with development (P11–37 vs. >P55), in males these responses significantly decreased with development. Our results suggest that the amplitude of 5-HT_1A_R-mediated currents in male *15q dup* mice may not be reduced and thus remain as large outward currents during adulthood. To validate this notion, further investigations using a range of mouse models of developmental disorders at various developmental stages are warranted.

The functional differences in 5-HT_1A_R-mediated outward currents between genotypes could arise from several molecular mechanisms. The first possible mechanism relates to expression levels of 5-HT_1A_Rs. However, similar receptor expression levels were seen in the WBS mouse model, and the gene expression level of 5-HT_1A_R in the *15q dup* mouse model was comparable to the WT mice in various brain regions, including the cortex [5]. Another possible mechanism may be functional enhancement of GIRK channels, which is a down-stream effector of 5-HT_1A_R activation, although the extent of baclofen-induced current amplitude was not different between genotypes, as shown in Fig. 2b. Thus, the mechanism underlying enhancement of 5-HT_1A_R-mediated inhibitory action appears to be receptor-specific. Our findings are in accordance with observations involving the mouse model of WBS [11]. Based on the neurochemical aspects, it is possible that the physiological receptor functions may be altered by extracellular concentrations of their transmitters, due to compensatory mechanisms [30]. In fact, the levels of 5-HT and its metabolite, 5-hydroxyindoleacetic acid (5-HIAA), were decreased in several brain regions including the PFC in the *15q dup* mouse model [2]. In contrast, the WBS model mice exhibited significant elevation of 5-HIAA, but a non-significant level of 5-HT in the PFC [10]. These opposite neurochemical characteristics in the brain probably contribute to each behavioral phenotype, such as anxiety and social behaviors, while the possibility for alteration of 5-HT_1A_R function by 5-HT levels could be ruled out. Our latest study revealed that L5 pyramidal neurons in the PFC shifted toward the excitatory state in the *15q dup* mouse model [18]. Therefore, the functional enhancement of 5-HT_1A_R might be compensating for the excitatory/inhibitory imbalance of pyramidal neurons in the *15q dup* mouse model.

Despite exhibiting larger 5-HT_1A_R-mediated outward currents in the *15q dup* model, the extent of 8OH-DPAT-induced inhibitory action of NMDAR-mediated currents was comparable to that of the WT group (Fig. 3). It is well-known that hypofunction of NMDAR-mediated synaptic transmission is involved in cognitive deficits [31] and social behaviors [32, 33]. Activation of 5-HT_1A_Rs reduces the surface NR2B level via the mechanism of microtubule dynamics, which is regulated by Ca^2+^/calmodulin-dependent protein kinase II (CaMKII) and the extracellular signal-regulated kinase (ERK) signaling pathway [12]. Since the down-regulation of CaMKII and ERK was due to the inhibition of adenylyl cyclase and subsequent protein kinase A by α subunit of G_i/o_, the 5-HT_1A_R-mediated modulatory action on NMDARs appears to be independent on eliciting GIRK channel-mediated outward currents. GIRK channel activation is regulated not only by βγ-complex of G protein subunits, but also signaling lipid, phosphatidylinositol 4,5-bisphosphate (PIP_2_) and intracellular Na^+^ ions [34]. Based on this mechanistic evidence, the local environment involving PIP_2_ and Na^+^ ion levels around 5-HT_1A_Rs might be altered in developmental disorders such as *15q dup* and WBS. Alternatively, since Src family tyrosine kinase activities regulate and suppress the 5-HT_1A_R-mediated GIRK channel activities in the PFC [35], these tyrosine phosphorylation states may be weakened in animal models. Therefore, further studies are needed to clarify the regulators of GIRK channel signaling and its upstream signal transduction system in animal models of developmental disorders.

## Supplementary information

**Additional file 1: Figure S1.** Activation of GIRK currents by 5-HT and baclofen in the prefrontal cortex L5 pyramidal neurons of WT male mice. A and B, 5-HT- and baclofen-induced currents were recorded by applying voltage ramps (− 140 to 0 mV, 500 ms) in the presence of 10 mM extracellular K^+^ in order to elicit K^+^ currents. Voltage-gated Na^+^ and Ca^2+^ currents and outward Ca^2+^-activated K^+^ currents were eliminated by simultaneous bath perfusion of TTX (0.5 μM) and cadmium (100 μM). *Left panels*: Current-voltage (I-V) relationships determined before (black traces) and after the application of agonists (red traces) by a constant voltage ramp command (upper trace). *Right panels*: agonist-sensitive currents were obtained by subtracting the current during the application of 5-HT (A) and baclofen (B) from the baseline shown in the *Left panels*. Under these conditions, 5-HT and baclofen induced currents that displayed .pronounced inward rectification and reversed polarity near the calculated equilibrium potential of − 68.9 mV (5-HT: − 61.6 ± 1.3 mV, *n* = 5; baclofen: − 64.0 ± 2.1 mV, n = 5) for a current carried by K^+^ ions. C and D, the effects of non-selective GIRK channel blocker, barium, on both 5-HT- and baclofen-induced outward currents. All recorded pyramidal neurons were Sag(+)-type neurons. The membrane currents were recorded at a holding potential of − 60 mV in the presence of 0.5 μM tetrodotoxin, in order to block spontaneous synaptic events. (C) Representative recordings of 5-HT-induced outward current pre- and post-barium treatment (Ba^2+^, 200 μM) and washout (> 15 min) in L5 pyramidal neurons. *Right panels*: Percent changes in the 5-HT-induced outward current amplitude. Ba^2+^ largely blocked the amplitude of the outward currents, which was partially recovered following washout (Ba^2+^: 26.6 ± 3.6%; Washout: 76.2 ± 7.0%, n = 5). (D) Representative recordings of baclofen-induced outward current pre- and post-Ba^2+^ treatment and washout (> 15 min) in L5 pyramidal neurons. *Right panel*: Percent changes in the baclofen-induced outward current amplitude. The baclofen-induced outward currents were also largely blocked by Ba^2+^ (Ba^2+^: 22.9 ± 1.7%; Washout: 78.5 ± 9.2%, *n* = 8).

## Data Availability

Please contact the author for data and materials requests.

## Abbreviations

5-HIAA: 5-hydroxyindoleacetic acid
5-HT: Serotonin
5-HT_1A_ receptor: 5-HT_1A_R
8-OH-DPAT: (±)-8-Hydroxy-2-(dipropylamino) tetralin hydrobromide
ACSF: Artificial cerebrospinal fluid
ASD: Autism spectrum disorder
CNQX: 6-cyano-7-nitroquinoxaline-2,3-dione
CaMKII: Ca^2+^/calmodulin-dependent protein kinase II
D,L-APV: D,L-2-amino-5-phosphonopentanoic acid
DRN: Dorsal raphe nucleus
EPSC: Excitatory postsynaptic current
ERK: Extracellular signal-regulated kinase
GIRK: G-protein coupled inward rectifying potassium
HCN: Hyperpolarization-activated cyclic nucleotide-gated cation
NMDAR: N-Methyl-D-aspartate receptor
NMDG: N-Methyl-D-glucamine
PFC: Prefrontal cortex
PIP_2_: Phosphatidylinositol 4,5-bisphosphate
SEM: Standard error of mean
WBS: Williams-Beuren syndrome
