# Supplementary material for: Upregulated 5-HT1A receptor-mediated currents in the prefrontal cortex layer 5 neurons in the 15q11–13 duplication mouse model of autism
Source: Mol Brain. 2020 Aug 24;13:115. doi: 10.1186/s13041-020-00655-9 (PMC7444243; doi:10.1186/s13041-020-00655-9)
Supplement: Supplementary file 1 — Additional file 1: Figure S1. Activation of GIRK currents by 5-HT and baclofen in the prefrontal cortex L5 pyramidal neurons of WT male mice. A and B, 5-HT- and baclofen-induced currents were recorded by applying voltage ramps (− 140 to 0 mV, 500 ms) in the presence of 10 mM extracellular K+ in order to elicit K+ currents. Voltage-gated Na+ and Ca2+ currents and outward Ca2+-activated K+ currents were eliminated by simultaneous bath perfusion of TTX (0.5 μM) and cadmium (100 μM). Left panels: Current-voltage (I-V) relationships determined before (black traces) and after the application of agonists (red traces) by a constant voltage ramp command (upper trace). Right panels: agonist-sensitive currents were obtained by subtracting the current during the application of 5-HT (A) and baclofen (B) from the baseline shown in the Left panels. Under these conditions, 5-HT and baclofen induced currents that displayed .pronounced inward rectification and reversed polarity near the calculated equilibrium potential of − 68.9 mV (5-HT: − 61.6 ± 1.3 mV, n = 5; baclofen: − 64.0 ± 2.1 mV, n = 5) for a current carried by K+ ions. C and D, the effects of non-selective GIRK channel blocker, barium, on both 5-HT- and baclofen-induced outward currents. All recorded pyramidal neurons were Sag(+)-type neurons. The membrane currents were recorded at a holding potential of − 60 mV in the presence of 0.5 μM tetrodotoxin, in order to block spontaneous synaptic events. (C) Representative recordings of 5-HT-induced outward current pre- and post-barium treatment (Ba2+, 200 μM) and washout (> 15 min) in L5 pyramidal neurons. Right panels: Percent changes in the 5-HT-induced outward current amplitude. Ba2+ largely blocked the amplitude of the outward currents, which was partially recovered following washout (Ba2+: 26.6 ± 3.6%; Washout: 76.2 ± 7.0%, n = 5). (D) Representative recordings of baclofen-induced outward current pre- and post-Ba2+ treatment and washout (> 15 min) in L5 pyramidal neurons. Ri [file 13041_2020_655_MOESM1_ESM.pdf]

Supplementary Figure 1

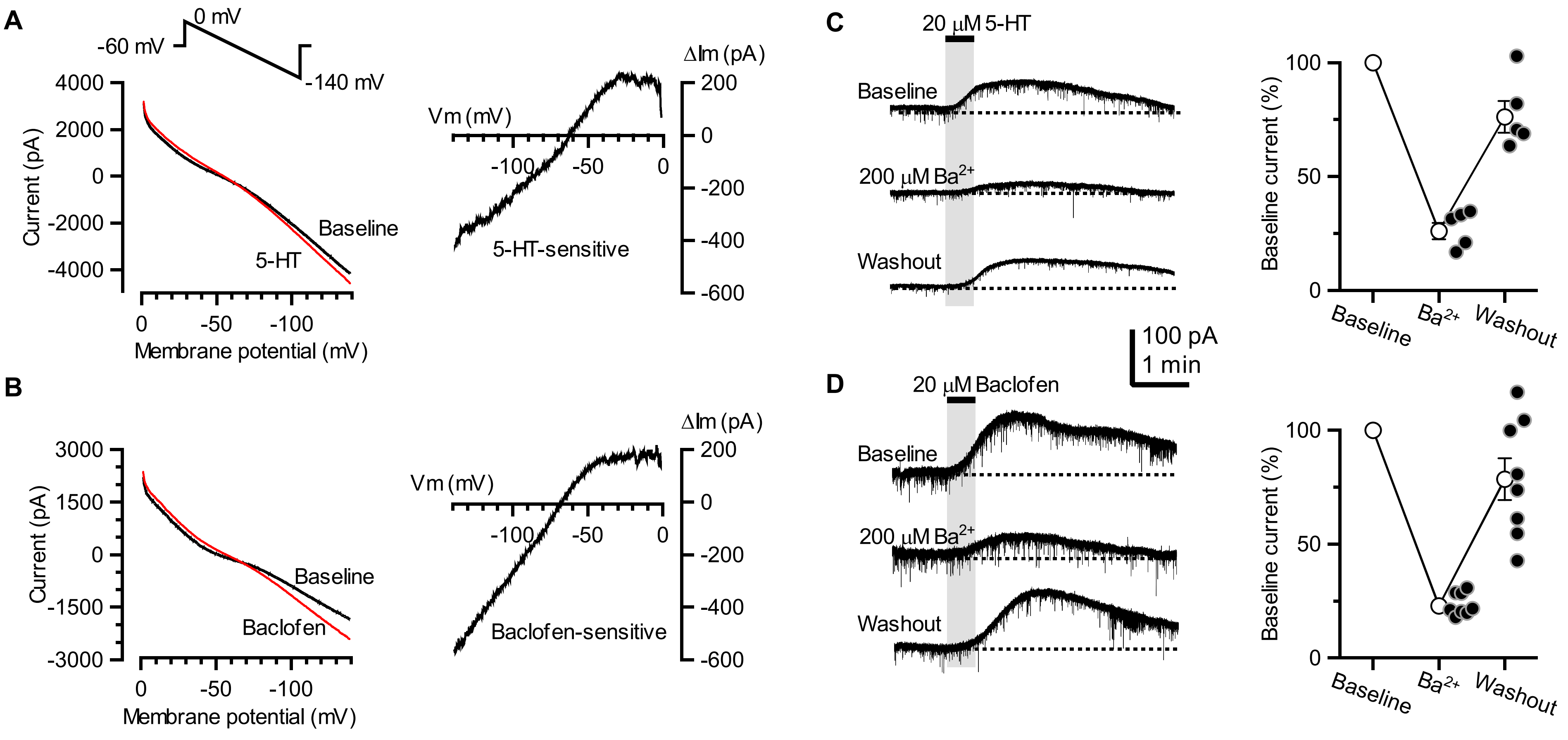

Supplemental Figure 1

Activation of GIRK currents by 5-HT and baclofen in the prefrontal cortex L5 pyramidal neurons of WT male mice. **A** and **B**, 5-HT- and baclofen-induced currents were recorded by applying voltage ramps (-140 to 0 mV, 500 ms) in the presence of 10 mM extracellular K^+ in order to elicit K^+ currents. Voltage-gated Na^+ and Ca^{2+} currents and outward Ca^{2+}-activated K^+ currents were eliminated by simultaneous bath perfusion of TTX (0.5 μM) and cadmium (100 μM). *Left panels*: Current-voltage (I-V) relationships determined before (black traces) and after the application of agonists (red traces) by a constant voltage ramp command (upper trace). *Right panels*: agonist-sensitive currents were obtained by subtracting the current during the application of 5-HT (**A**) and baclofen (**B**) from the baseline shown in the *Left panels*. Under these conditions, 5-HT and baclofen induced currents that displayed pronounced inward rectification and reversed polarity near the calculated equilibrium potential of -68.9 mV (5-HT: -61.6 ± 1.3 mV, n = 5; baclofen: -64.0 ± 2.1 mV, n = 5) for a current carried by K^+ ions. **C** and **D**, the effects of non-selective GIRK channel blocker, barium, on both 5-HT- and baclofen-induced outward currents. All recorded pyramidal neurons were Sag(+)-type neurons. The membrane currents were recorded at a holding potential of -60 mV in the presence of 0.5 μM tetrodotoxin, in order to block spontaneous synaptic events. **(C)** Representative recordings of 5-HT-induced outward current pre- and post-barium treatment (Ba^{2+}, 200 μM) and washout (> 15 min) in L5 pyramidal neurons. *Right panels*: Percent changes in the 5-HT-induced outward current amplitude. Ba^{2+} largely blocked the amplitude of the outward currents, which was partially recovered following washout (Ba^{2+}: 26.6 ± 3.6%; Washout: 76.2 ± 7.0%, n = 5). **(D)** Representative recordings of baclofen-induced outward current pre- and post-Ba^{2+} treatment and washout (> 15 min) in L5 pyramidal neurons. *Right panel*: Percent changes in the baclofen-induced outward current amplitude. The baclofen-induced outward currents were also largely blocked by Ba^{2+} (Ba^{2+}: 22.9 ± 1.7%; Washout: 78.5 ± 9.2%, n = 8).
